# Peer-engagement and its role in reducing the risky behavior among crack and methamphetamine smokers of the Downtown Eastside community of Vancouver, Canada

**DOI:** 10.1186/s12954-016-0108-z

**Published:** 2016-06-08

**Authors:** Ehsan Jozaghi, Hugh Lampkin, Martin A. Andresen

**Affiliations:** BC Centre for Disease Control, 655 West 12th Avenue, Vancouver, V5Z 4R4 British Columbia Canada; Vancouver Area Network of Drug Users, 380 East Hastings Street, Vancouver, V6A 1P4 British Columbia Canada; School of Criminology, Simon Fraser University, 8888 University Drive, Burnaby, V5A 1S6 British Columbia Canada

**Keywords:** Peers, the Downtown Eastside, Crack, Methamphetamine, VANDU, HIV, Hepatitis C, Advocacy, People who smoke drugs

## Abstract

**Background:**

The role of peers (former or current drug users) in reducing risky behavior within methamphetamine and crack smokers has not been well described or researched. The current study not only explores the role of peers in reducing risk factors for morbidity within the illicit drug smoking population in the Downtown Eastside (DTES) community of Vancouver but it also investigates the changes in the nature of drug use after the closure of an unsanctioned smoking facility.

**Methods:**

The data pertain to qualitative interviews with 10 peers and 10 illicit drug smokers. The semi-structured interviews were conducted through community-based research, and the digital transcripts were analyzed via NVivo 10 software.

**Results:**

The results indicate that peers (former and current drug users who are employed as educators) are instrumental in transferring risk reduction knowledge within crack and methamphetamine smokers. For example, these peers have been able to teach users about the risk of sharing pipes, using brillo, and using public drug. Furthermore, the Vancouver Area Network of Drug Users provides employment for crack and methamphetamine users in Vancouver who tend to have scarce sources of employment. However, since the closure of the unsanctioned inhalation facility, there has been significantly more public drug use and pipe sharing in the vicinity of the facility, placing drug smokers at significant risk of arrest, violence, and blood-borne infections.

**Conclusions:**

The current study recommends expanding the harm reduction peer network for people who smoke illicit drugs in the DTES community of Vancouver who have historically been underserved.

## Background

Ample evidence suggests that inhaling crack cocaine and methamphetamine is not only on the rise in the urban areas of Canada but it also has replaced traditional problems such as intravenous drug use, specifically in Vancouver, Canada [2–5]. In fact, in comparison to other Canadian provinces, British Columbia has one of the highest prevalence of daily crack and methamphetamine usage [6, 7]. This is particularly problematic in Vancouver’s Downtown Eastside (DTES) community, where daily crack and methamphetamine use increased from 7.4 % in 1996 to 42.6 % in 2005 within a cohort of people who inject drugs [1].

Recent research has documented the severe health problems associated with smoking illicit drugs, especially crack and methamphetamine. While sharing crack and methamphetamine pipes has been routinely linked to the outbreak of respiratory illnesses such as tuberculosis (TB), research has also highlighted a plausible link to hepatitis C (HCV) and human immunodeficiency virus (HIV) transmission. Some researchers have postulated that blood-borne infections may be transmitted via pipes because many illicit drug smokers have sores, blisters, and cuts on their lips and oral cavities that are caused by contact with hot or broken glass pipes that are not heat resistant [8, 9].

Vancouver Coastal Health (VCH) in 2011 began distributing harm reduction smoking paraphernalia as part of their overall preventative program in the DTES community with over 4000 estimated crack and methamphetamine users [9]. Currently, the harm reduction smoking paraphernalia are distributed by the British Columbia centre for disease control at various locations in the DTES community such as single room occupancy hotels, the Washington needle depot, drug users resources centre, InSite, and Vancouver Area Network of Drug Users’ (VANDU).

**Fig. 1:**
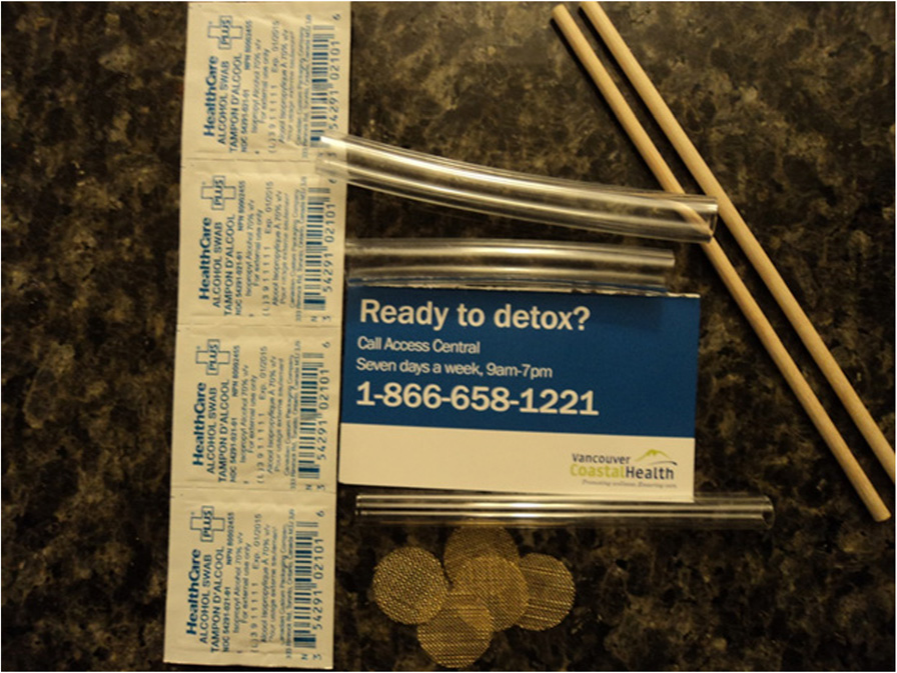
Crack pipe kits

Despite numerous studies showing the benefit of supervised smoking rooms (SSRs) in reducing harm and risky behavior in PWSDs, the local health authority and the city of Vancouver have shown little interest in applying for exemption under *the Controlled Drugs and Substances Act* (CDSA) [10–12]. However, to combat the growing health concern associated with illicit drug smoking, the city of Vancouver started distributing crack pipe kits for free in the DTES community [13] (Fig. 1).

**Fig. 2:**
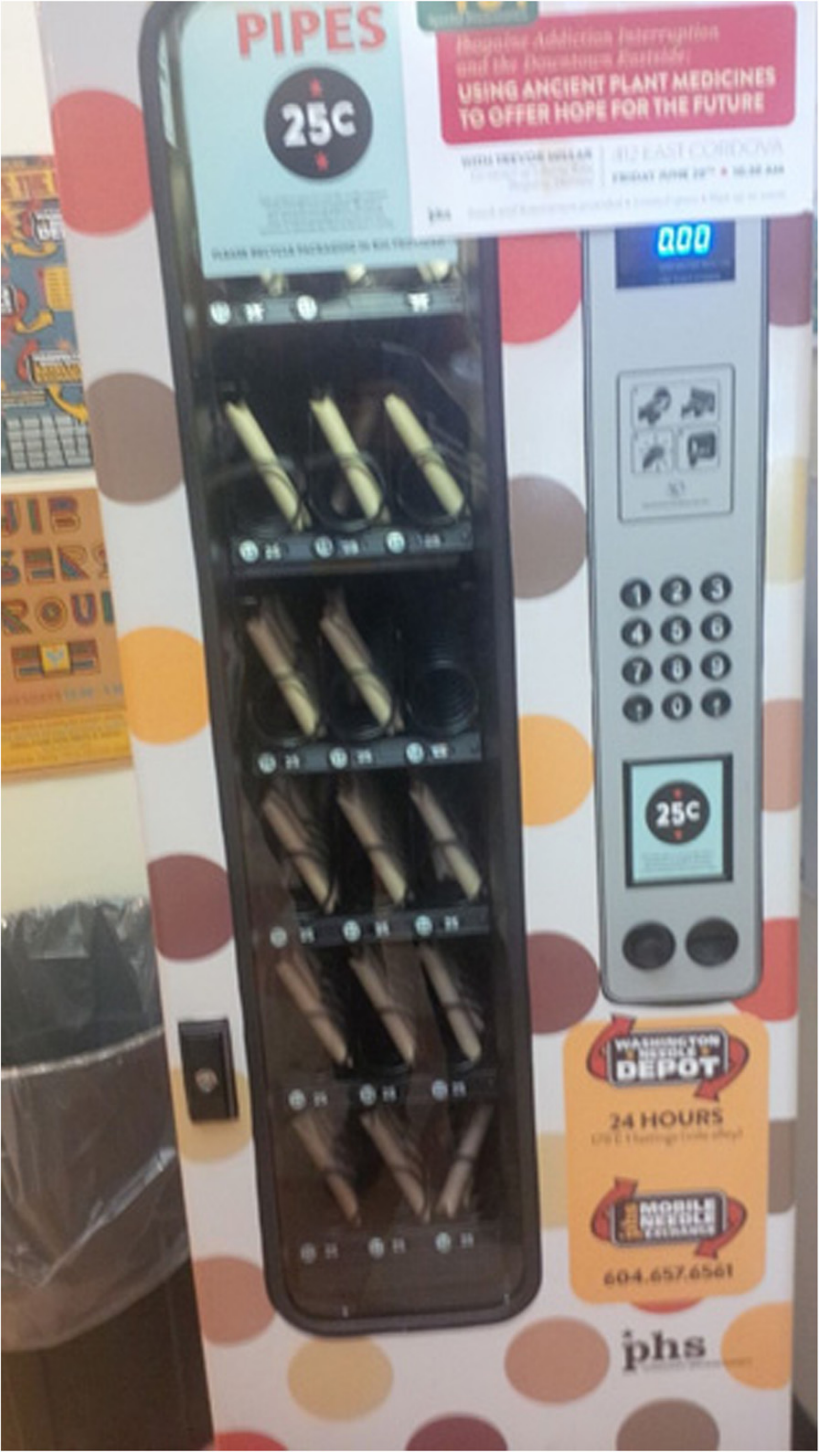
Pipes accessible through vending machines

Additionally, pipes were made accessible through vending machines in the DTES community by many non-profit organizations (Fig. 2).

However, research that evaluated the outcome of the pipe distribution program suggested that the impact of pipe distribution alone was limited to safe use practices and it did not address some of the root causes of violence and public drug use associated with illicit drug smoking [13]. Furthermore, studies conducted in this area have paid limited attention to the role of “peers” in transforming the behavior of illicit drug smokers.

Peer work has been shown to be successful in numerous setting (e.g., developing and developing courtiers) in combating HIV and risky behavior and improving the overall health ([14–16]). The peer work has also been extremely effective within people who inject drugs (PWIDs), sex workers, and marginalized youth populations ([17, 18]). This is particularly significant when the overall harm reduction delivery model in the DTES community has proven extremely successful when peers are employed in the distribution of harm reduction supplies [18–20].

This has been particularly true for a peer-driven drug user organization known as VANDU in Vancouver’s DTES community. VANDU brings together more than 1000 drug users from the area, encourages safe practices by giving drug users the opportunity and voice to design and implement harm reduction interventions that are meaningful to them through a democratic and dignified process. All members are encouraged to attend the weekly meetings and are encouraged to partake and shape the organization’s drug policy.

At all harm reduction locations in the DTES community, interventions are done by those members of the community who have experience in safe illicit drug practices (“peers”). For example, similar peer work has been implemented at various harm reduction location in the DTES community, including but not limited to the drug user resource centre (DURC), Washington’s needle depot, and InSite. The overall objective is to empower those who use drugs that will in return reduce the harms associated from drug use, for both the users, and their communities.

An example of such harm reduction initiative was opening the SSRs in the DTES community through an organic and democratic process by VANDU members. The unsanctioned SSR operated by peers was a bathroom. The illicit drug smoker who intended to use the bathroom informed the peer at the front desk about their intention to use the bathroom that contained a ventilation system. During the noted interactions, the illicit smoker could acquire harm reduction kit shown on Fig. 1 and ask for harm reduction education/help.

However, the peer-driven, unsanctioned SSR was shut down on December 2013 after the VCH through (VANDU’s funding agency) a formal meeting on 12 December 2013 and a formal letter through the executive director of VCH’s Vancouver community requested the unsanctioned peer driven facilities to cease operation because such activities contradict the CDSA [9, 12]. As a result, the current study not only explores the role of “peers” in reducing harm and educating PWSDs regarding safer smoking practices, but it also explores the changes in the nature of drug use in the vicinity of the unsanctioned SSRs in the DTES community after the unsanctioned location was directed to shut down.

## Methods

The first author who has been a researcher in the DTES community since 2009 and had volunteered and worked in the DTES community at various capacities since 2011 initially approached the VANDU’s Board of Directors on December 2013 regarding a potential study related to peer work and the closure of the SSR. After consultation and input in the study design and recruitment process by the board of directors, the study was approved on December 2014 by VANDU. The Office of Research Ethics at Simon Fraser University also approved the study. The recruitment began when VANDU’s Board of Directors appointed a research coordinator who worked closely with the ethnographer.

The research coordinator, who was the president of VANDU and had served as the executive board member for many years, initially started recruiting people who volunteered as peer educator at VANDU. After consultation with the VANDU board and the first author’s qualitative experience, it was decided to choose ten peers and ten drug users. Ten peers were selected by the research coordinator to partake in semi-structured interviews. Peers are current or former illicit drug users who are employed at various roles at VANDU in the implementation and design of the harm reduction program. The selection criteria for peer selection were based on three factors: (1) be a former or current illicit drug smoker; (2) volunteers on the weekly basis at VANDU or other harm reduction locations in the DTES community; and (3) knowledgeable on harm reduction philosophy.

The research coordinator also recruited ten individuals who are currently smoking illicit drugs. The selection criteria were based on three criteria: (1) have used the SSR in the past; (2) are currently known to be homeless or poorly housed in the neighborhood; and (3) are known to acquire their harm reduction smoking paraphernalia from VANDU. Housing was used as one of the main criteria’s to help in conceptualization of risk associated with public drug use for people who do not have the luxury of smoking their illicit drugs at a private dwelling.

The research coordinator was paid an honorarium of CAN$5 for each referral. The participants who, after reading the consent statement, agreed to partake in the semi-structured interviews, by giving a verbal consent, were paid CAN$20 after the interview. All the names used in this research are pseudonyms. Semi-structured interviews were conducted in a VANDU office with a digital tape recorder enhancing the interview process by allowing the researcher to write keywords and phrases in his notebook for later analysis and allowing the collected data be typed at a later date. The first author spent 8 h of observation in and around the vicinity of VANDU (two block area) by walking the alleys and parking lots in the DTES community for a period of 2 weeks on different days and times to confirm the narratives from the peers and illicit drug smokers that public crack or methamphetamine drug use has significantly increased.

An interview guide based on ground theory in qualitative research was used during the semi-structured interviews where the interview guided the research but did not intend to be either static or confining. The interview guide helped the discussion around the role of peers as educators, support around harm reduction behavior, and initiatives in the DTES community. Moreover, additional lines of inquiry were formed after observations around the vicinity of VANDU such as the sharing behavior, violence, safety, police, and accessibility of a safe place. Interviews on average lasted for 35 min, with the longest interview well over an hour and the shortest interview just under 20 min. Different topics were used for peers and people who were not peers. Peer topics focused on the history of the harm reduction paraphernalia, their role in education, and advocacy. The topics for people who were not peers focused on their public drug use, the changes in their behavior since the closure of the inhalation facility and the role of peers in reducing their risky behavior.

Each interview was transcribed verbatim. The coding and analysis was an iterative process and began shortly after the first few interviews by the first author. NVivo qualitative software (version 10) was used to facilitate coding after all the transcribed interviews, that did not contain any identifier information, were imported into the software. Initially, an inductive inquiry using the NVivo version 10 was implemented. The process of coding through the inductive process according to Fereday and Muir-Cochrane [21] “involve recognizing (seeing) an important moment and encoding it (seeing it as something) prior to a process of interpretation” (p. 83). Boyatzis [22], for example, defined a theme as “a pattern in the information that at minimum describes and organizes the possible observations and at maximum interprets aspects of the phenomenon” (p. 161). Word recognition of NVivo 10 feature was implemented to enhance the inductive coding process and to identify the most frequently spoken words or phrases.

In addition to the inductive approach described in Boyatzis [22], in our analysis of the text in this study, we also used a deductive approach, as described in Crabtree and Miller’s [23] study. This deductive approach consisted of a guide in the form of codes from a code selection to be applied as a means of organizing digital transcripts for subsequent interpretation using NVivo version 10. When using a pre-existing guide/template, the first author who conducted the interviews defined the template (or codebook) before starting an analysis of the transcript via NVivo software version 10.

The template according to Fereday and Muir-Cochrane [24] is most often based on a preliminary search/scanning of the transcript, but in this study, the pre-existing guide was developed not only on theoretical framework and research questions for both peers and drug users but also the search for the most frequent words and phrases. At a later opportunity, the highest recorded words or phrases were highlighted as a theme in the inductive coding process where latent meaning or themes were being identified. In other words, the first author allowed research findings to emerge from “the frequent, dominant, or significant themes inherent in raw data, without the restraints imposed by structured methodologies” ([24], p. 238). The two participant group transcripts were used together in the analysis. To ensure validity, the emerging themes and results were presented to the VANDU Board of Directors at their board meetings.

## Results

Overall, 20 participants gave their oral consent to have their semi-structured interviews recorded. The sample included 6 females and 14 males. The average age of the participants was 45, with 12 of interviewees self-identifying as belonging to members of visible minority (e.g., Indigenous peoples and African Canadians). All the participants resided in the DTES community and their income generation avenues where from the government (e.g., social assistance, pension or disability income) or volunteer stipends. All the participants have resided in the DTES community for more than 5 years.

The first author with collaboration with the second author and the board of directors reached the targeted population set out in the study design. However, the goal of qualitative research generally is not to reach a generalized sample, rather the goal is to find rich and detail description of the phenomena being investigated via small, sometimes unrepresentative sample [25]. The finding section below is excerpt using direct quotation and narrated observations from both participant groups using both inductive and deductive method of analysis from peers and drug users.

### Harm reduction supplies

Our analysis points to the scarce harm reduction supplies and restricted access to good quality pipes up until early 2011. According to Robert (peer), this caused damage to the face, lips, and mouth of the PWSDs:

There were selling eye dropper back then, very thin glass at grocery stores for five dollars. So you would be feeding your rock and the rock would be heated so much, the Brillo would be heated so much that the class would blow up. They would literally blow up. Your eyes would be damaged your lips your nose, your whole face would be damaged with hot glass. Then they came up with the love flowers, where they would have stemmed glass that has plastic flowers inside. But the problem with those glasses were the same it wasn’t pyrex. They claimed it was pyrex but it wasn’t. These corners stores and grocery stores claim that these “love flowers” as they called them back then were pyrex; it would blow up in your face. If you didn’t notice it carefully, and you started to use a push sticks and didn’t use a mouth piece and then you are cutting your lips. It was ridiculous it was very hard to get proper glass back then.

Furthermore, the high prices of pipes and other related items made it very challenging for PWSDs to access harm reduction supplies. Many of the related items were inaccessible or were too expensive, encouraging many users to engage in unhealthy behavior such as sharing. For instance, according to Joe (peer):

It was hard. People had to pay a lot of money for pipes. Back then people sometimes paid upwards of $20 for a pipe especially on welfare day. I remember people were stealing Brillo from grocery stores and getting caught. A lot of stuff there were selling back then was unsafe. Because people were sharing each other’s pipes, because they couldn’t get what they needed like Brillo, pipe and lighter.

To combat the growing harm that many drug users faced as result of lack of available harm reduction supplies, VANDU’s Board of Directors decided to purchase heat resistant pipes and distribute the noted items at purchased cost. Later in 2011, according to Max (peer), Vancouver Costal Health decided to award a contract to VANDU so they could assemble the noted items in their office:

So VANDU through Vancouver coastal health started to hand out pyrex glasses that were heat resistant and had mouth pieces, pushes sticks, alcohol swap, and even some information regarding how to get to detox and they also had heat resistant filters rather than the crap you could find in the grocery stores.

### Risky behavior

According to Joe (illicit drug smoker), the process of crack or methamphetamine drug use is a complicated process:

On the welfare day especially people are selling crack pipes for $20 … So normally what happen [is that] people will have money to buy their crack or side (methamphetamine) but they don’t have enough money to buy a pipe so the buddy lend a pipe so he would want to have his pipe back so he can smoke the residue. People don’t mind giving up their pipes so they can have left over toke after the person is done. To do that, I would transfer the germs and diseases back and forth. Nowadays pipes are free most of the times but people are still doing sharing.

The complicated process highlighted by Joe was the main reason behind VANDU’s Board of Directors turning their bathroom in the main building into a smoking facility in late 2011. This small facility, according Michelle (illicit drug smoker), is a safe place for many vulnerable drug users who would like to escape the violence on the street:

People were getting mugged, robbed for a pipe. There were stabbing going on in the back alleys back then before we started the inhalation program. It was pretty ridiculous out there back then just for one piece of glass. I had two friends that were actually a stabbed for their pipes. So they were basically homeless and I was working at the bottle depot on the Hastings streets at the time and they were behind the alley by the bottle depot and one of them came running into the bottle depot with a knife still in him in his stomach. I told him what happened? And he said I got stabbed for my pipe! So we had to call an ambulance. The other guy got slashed across his chest for his pipe. So stabbed in the stomach for one person that I know and slashed across the chest for another person for just a piece of glass.

Moreover, the noted facility could have enhanced safety if users choose to smoke their illicit drug at the SSR. For example, as Bryan (illicit drug smoker) puts it:

So after you smoke your pipe it becomes a very hot piece of instrument so they would see the police coming in the alley, they know that the police have seen them smoking their crack pipe. And they place that hot piece of metal or glass into their pocket. So now what’s happening is that the pipe is burning their clothes and their skin. So they’re doing that so they could hide the piece of glass. So I remember one guy at the bus stop had to hide his crack pipe from the cops he puts it into his back pocket and sat on it. It melted his pants and the skin and he was bleeding because it hit the major artery. The police ended up calling the ambulance.

So the small inhalation room was not only effective in reducing the sharing behavior and violence but it also served as a safe place where PWSDs can relax and smoke their illicit drugs without being harassed by the police or people on the streets.

### Closure

All the ten peers believed that their work toward establishing this inhalation facility has had a significant impact on the culture of illicit drug use and the micro-environmental factors that has brought disproportionate risk factors toward the drug users. For instance, as Robert (peer) describes:

So for a lot of people they just can’t smoke their crack outside because they just get paranoid and they lose their mind. Will certainly need … inhalation room so that people can come inside and they won’t bother anybody and they can be monitored. Being in an alley to inhale your drug is not only degrading and demeaning but it’s also pure dangerous.

However, the closure of unsanctioned inhalation room, according to Neil (peer), had a significant negative effect in the vicinity of VANDU:

After they forced VANDU to close the inhalation room, you would see rows and rows of people smoking their dope outside behind the alleys right by VANDU. Early in the morning you could see people sharing pipes, it was like going back to early years in the Downtown Eastside … People actually came to our office begging to use the bathroom again for smoking… So the Vancouver coastal health by shutting down the inhalation room sent us back a decade on harm reduction policy, everything that VANDU had worked for over the past few years was just down the drain. So alleys were crowded, doorways overcrowded, people are basically crouched in doorways paranoid and scared.

Many participants indicated that after the closure, they were forced to go back into the alleys. In the streets around VANDU after the facility was shut down, many of the PWSDs could be seen leaning against the garbage bins or back door entrances not only hiding from the elements but also unwanted visitors. George (illicit drug smoker) reiterated that “people, come and approach you and see what you got, and try to mooch off you. Or rip you off trying to sell you bonk and stuff like that. [what is bonk?] Bonk is the stuff that may look like cocaine or may look like crack but it isn’t.”

### Education

Before, during, and after the closure of the unsanctioned facility, VANDU has been actively involved in providing weekly meetings, education workshops, marches, and advocacy on behalf of marginalized drug users. According to Linda (peer), this is particularly important because this form of peer work served to educate PWSDs:

We saw a lot of people come to VANDU because we offer the free crack pipe kits. People used to come in and asked us to make the crack kit for them. We as peers tell people what the safest way to use rock and crystal is. So we tell people where to buy their rock and side, we suggested to them some areas that were safe and the quality of the crack were good because sometimes when people been working all day long, to get their money for their rock, and then they go to see somebody and that person bunks them. And also don’t be in dark alleyways by yourself. Don’t ever ever use alone in an alley.

The peers teach PWSDs not only where to purchase their drug, but peers like Adam (peer) teach the drug users about safer use through their meetings and interactions at VANDU. One such interaction, according to Adam (peer), relates to using screens:

I’ve known two people that had to go to the hospital because they did not have the proper Brillo and the screen. So they basically sucked in the Brillo into their throat. The Brillo basically gets red hot and when you inhale that into your throat it burns your lungs … So when you burn the Brillo first you would see a lot of black smoke coming out of it. So basically it can’t be healthy for you when you constantly sucking on that Brillo. And a lot of people don’t end up preparing their stuff properly. But these screens that now we provide at VANDU they don’t break up as easily as the Brillo you would buy at the grocery store. So they’re not as easy to inhale as a regular Brillo. So we tell people how to break down their screen so it would not break down or harm them.

However, the outreach and education that many peers provide at VANDU goes beyond the simple harm reduction help. In fact, based on Ashley’s (peer) account, VANDU also provides employment and volunteer work where many PWSDs feel they are part of something bigger:

It’s amazing to see how many people that smoke rock, learn things new. So our peers that handouts crack pipes usually have interactions with users and they teach people showing different ways to put their screens properly … So I know people that now because of our education have learned how to properly set of their crack pipes … For example it is amazing to see more people are now using this screens rather than using Brillo. Also another big difference is that we taught people to use mouth pieces when they are sharing their crack pipes and you would see that people are actually using those mouth pieces on daily basis. We also provide volunteer stipend work for drug users to build a crack kit which contain information for detox. The crack kit also contains screens, a pipe, alcohol wipes and two mouth pieces.

Research indicates that PWSDs are able to change their behavior when they are treated with care, dignity, and appropriate education [10]. A number of research studies have also highlighted the potential role of peer run organizations in transforming the drug behavior and culture of illicit drug use [14, 15, 17, 18].

## Discussion

In summary, our findings underscore the critical role that peer-run drug user organizations, peer education, and peer work can do for a very marginalized illicit drug-using population. The peers were able to transform many risk factors that historically had placed PWSDs at significant risk of blood-borne and respiratory infections such as HIV, HCV, and TB. In addition, the results of this study demonstrate that drug user advocacy groups have been instrumental in improving the health and social well-being of PWSD, transforming the risky smoking behavior of the clients who used the SSR. Moreover, peer-led organizations have given a voice to the most vulnerable members of society who otherwise would not be represented in forums such as weekly meetings, social mobilization, and advocacy. Results in the current report signify the critical role that a peer-led organization can play in creating new, safe, non-judgmental places and welcoming locations.

Finally, a controversial harm reduction program in the DTES community has provided new insight into effectiveness of harm reduction programs, more generally. The unsanctioned inhalation facility operated by VANDU for a few years reduced public illicit smoking, sharing, and violence. In addition, the unsanctioned SSR improved the overall safety and health of all the participants who relied on the room on the daily basis with some of the clients eventually using other services. Some of the clients were even able to acquire work and/or volunteer positions in the organization. The result of this research points to the devastating consequences of shutting the noted location where the public drug use, violence, and risky behavior increased immediately after the closure in the vicinity of VANDU.

Jozaghi [9] demonstrated that the noted unsanctioned inhalation facility had reduced 55 cases of HCV every year on average during the very short operation. McNeil et al. [12] also demonstrated that inhalation facilities such as the unsanctioned one operated by VANDU had the potential to improve the health and safety of the illicit drug smoking population. The findings in the current study when taken as whole points to the effectiveness of harm reduction programs for illicit drug smokers. It is well known that city, provincial, and federal governments spend more money on policing and drug enforcement than the harm reduction programs [26, 27]. For instance, harm reduction programs for illicit drug users in 2004–2005 received a fraction of funding earmarked for the total funding marked for drug use services (e.g., 3 % only) [26]. This level of funding is particularly worrisome when compared to ever increasing law enforcement funding that has been linked to increased harm and violence in the drug market, especially increases in risky injection behavior among PWIDs [28].

However, this study has demonstrated that employing former and current illicit drug users—through small stipends and funding harm reduction peer-driven organization—could have influences on the risky behavior of the people who use drugs. Such programs, such as expanding the pipe program, have the potential to reduce many of the well-known risk factors described in this study. Moreover, a peer-run SSR has the potential to give a marginalized population a space where they feel empowered to advocate for their rights and issues that may affect their health and well-being. This is very important in the realm of drug policy in Vancouver because such activism on behalf of PWSDs by drug users themselves has broken down many boundaries that have previously brought disproportionate suffering for illicit drug users.

The VCH and the various levels of the governments have the opportunity to change their policies and legislations so they can be aligned by the best and updated knowledge while simultaneously providing harm reduction and preventative services by supporting the marginalized members of society. Policy makers should start considering providing free smoking harm reduction paraphernalia equipment through peers. Supervised inhalation facilities should also be considered as an expansion of a health-care program. The government and health agencies must move toward a policy where the access to free harm reduction smoking paraphernalia and supervised inhalation facilities are given the same priority as other urban health problems such supervised injection facilities. As this study has recounted the lived stories of many PWSDs, harsher and stricter policies toward drug users will make the overall health of this population much worse because it will increase discrimination and risky behavior and make health-care delivery more difficult.

## Conclusions

The rate of blood-borne infections within PWID population was not reduced because of more emphasis on strict policies; rather, it was attributed to more relaxed polices that recognized that harm reduction should be given more attention [29–32]. Similarly, as this study has shown, treating PWSDs with more dignity, kindness, and empathy through increasing harm reduction supplies via peer drug users will improve their overall quality of life. Programs such as a peer-driven inhalation facility will reduce crime and improve the health and social tenure of PWSDs because addiction is viewed as a health issue rather than a criminal justice or public nuisance. When PWSDs are connected to their peers, they will begin to ask for harm reduction practices and social services and slowly improve their well-beings.

This will ultimately help in conceptualizing the illicit drug smokers not as a public health nuisance, rather as people who need immediate help and attention through more effective harm reduction policies that will include supervised inhalation rooms operated by peers. There are numerous limitations associated with the study such as generalizability of the research in the vicinity of VANDU to the greater DTES community. Also, the reliability of memory since the study was conducted after a year the facility was forced to shut down could have significant effect on the authenticity of the narratives. However, since the stories of participants were reinforced by the board of directors, such influences are deemed to minimum. Future studies need to investigate through quantitative and social networks the role of peer harm reduction networks on the overall risk reduction of PWSDs.

## Abbreviations

CDSA, Controlled Drugs and Substances Act; DTES, Downtown Eastside; DURC, drug user resource centre; HCV, hepatitis C virus; HIV, human immunodeficiency virus; PWIDS, people who inject drugs; PWSD, people who smoke drugs; SSR, supervised smoking room; TB, tuberculosis; VANDU, Vancouver Area Network of Drug Users; VCH, Vancouver Coastal Health
